# Iatrogenic Parasitic Leiomyoma in the Lower Right Abdomen: A Rare Case in Plastic Surgery

**DOI:** 10.7759/cureus.65924

**Published:** 2024-08-01

**Authors:** Maako Fujita, Toshihito Mitsui, Sakurako Kunieda, Michika Fukui, Natsuko Kakudo

**Affiliations:** 1 Department of Plastic and Reconstructive Surgery, Kansai Medical University, Hirakata, JPN

**Keywords:** subcutaneous tumor, lower abdomen, myomectomy, morcellator, iatrogenic parasitic leiomyoma

## Abstract

A parasitic leiomyoma has no connection with the uterus itself and obtains its blood supply from the surrounding tissues. A parasitic leiomyoma that develops iatrogenically is called an iatrogenic parasitic leiomyoma. Clinical reports on parasitic leiomyoma are common in gynecology but not in plastic surgery. We report a rare case of an iatrogenic parasitic leiomyoma in a 46-year-old woman who was referred to our plastic surgery department. She presented with the main complaint of a subcutaneous mass in the lower right abdomen and had a history of laparoscopic myomectomy eight years ago. Magnetic resonance imaging showed two masses in the subcutaneous tissue of the lower right abdomen and the posterior rectus abdominis. The excision of these masses was performed through an abdominal wall incision. Histopathological examination revealed that the extracted mass was a leiomyoma. Plastic surgeons must keep in mind the appropriate management of rare cases of parasitic leiomyoma.

## Introduction

Parasitic leiomyoma can originate from a subserous pedunculated myoma, has no connection with the uterus itself, and obtains its blood supply from the surrounding tissues [1]. Although the detailed mechanisms involved in the occurrence and development of parasitic leiomyoma are not clear, parasitic leiomyomas are further classified as primary spontaneous and secondary parasitic myomas [2]. A parasitic leiomyoma that develops iatrogenically is called an iatrogenic parasitic leiomyoma [3]. Iatrogenic parasitic leiomyoma probably occurs from the dissemination of uterine tissue by myomectomy with morcellation [3,4]. Its incidence has been reported to range from 0.12% to 0.95% when a morcellator is used in laparoscopic myomectomy [5]. The frequency is very low when morcellators are not used. However, the actual frequency may be higher because asymptomatic iatrogenic parasitic leiomyomas are sometimes detected during regular checkups [4]. Morcellators are advantageous because their minimally invasive nature allows tissue removal through small skin incisions. However, if intraperitoneal leiomyoma debris is dispersed into the abdominal cavity during morcellation, an iatrogenic parasitic leiomyoma can develop [6]. In 2014, the U.S. Food and Drug Administration (FDA) discouraged the use of laparoscopic power morcellators during myomectomy because they can spread malignant uterine sarcoma cells [7]. This alert should be considered important for non-malignant uterine tissue extraction during laparoscopic surgery. Management of a parasitic leiomyoma is commonly challenging for treating physicians in gynecology and obstetrics departments [3,8]. Since the etiology is unknown [6,9], parasitic leiomyoma must be considered in the differential diagnosis of every case of abdominal mass. We present a rare case of parasitic leiomyoma presenting as a subcutaneous mass in the lower abdomen of a Japanese woman with a previous history of morcellation myomectomy.

## Case presentation

 A 46-year-old Japanese woman was referred to our plastic surgery department complaining of a subcutaneous mass in the lower right abdomen. She did not suffer from pain. Distention was not externally detected, but a mass with a diameter of 1 cm was found in the right lower abdomen by palpation. Hematological examinations, including white blood cells (7,100/μL) and markers of hepatic and renal function, were normal. Her inflammation marker was normal (CRP: 0.02 g/L). An ultrasound examination at the previous hospital suggested a benign tumor in the subcutaneous fat layer, and she was referred to our hospital in 2022. The patient had undergone laparoscopic myomectomy for uterine fibroids, which were extracted by a laparoscopic power morcellator in February 2014.

**Figure 1:**
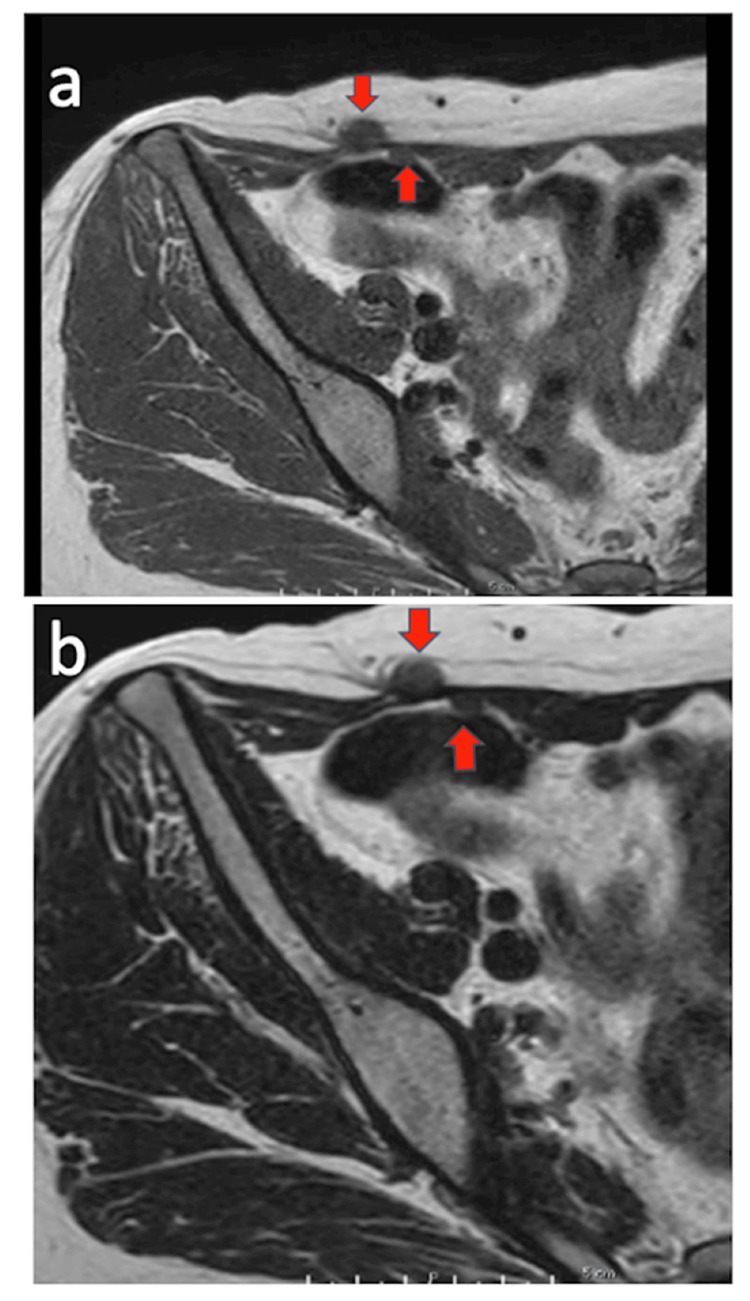
Magnetic resonance image (MRI) of the right abdomen. (a) T1-weighted axial image. (b) T2-weighted axial image. Arrows show the position of masses.

**Figure 2:**
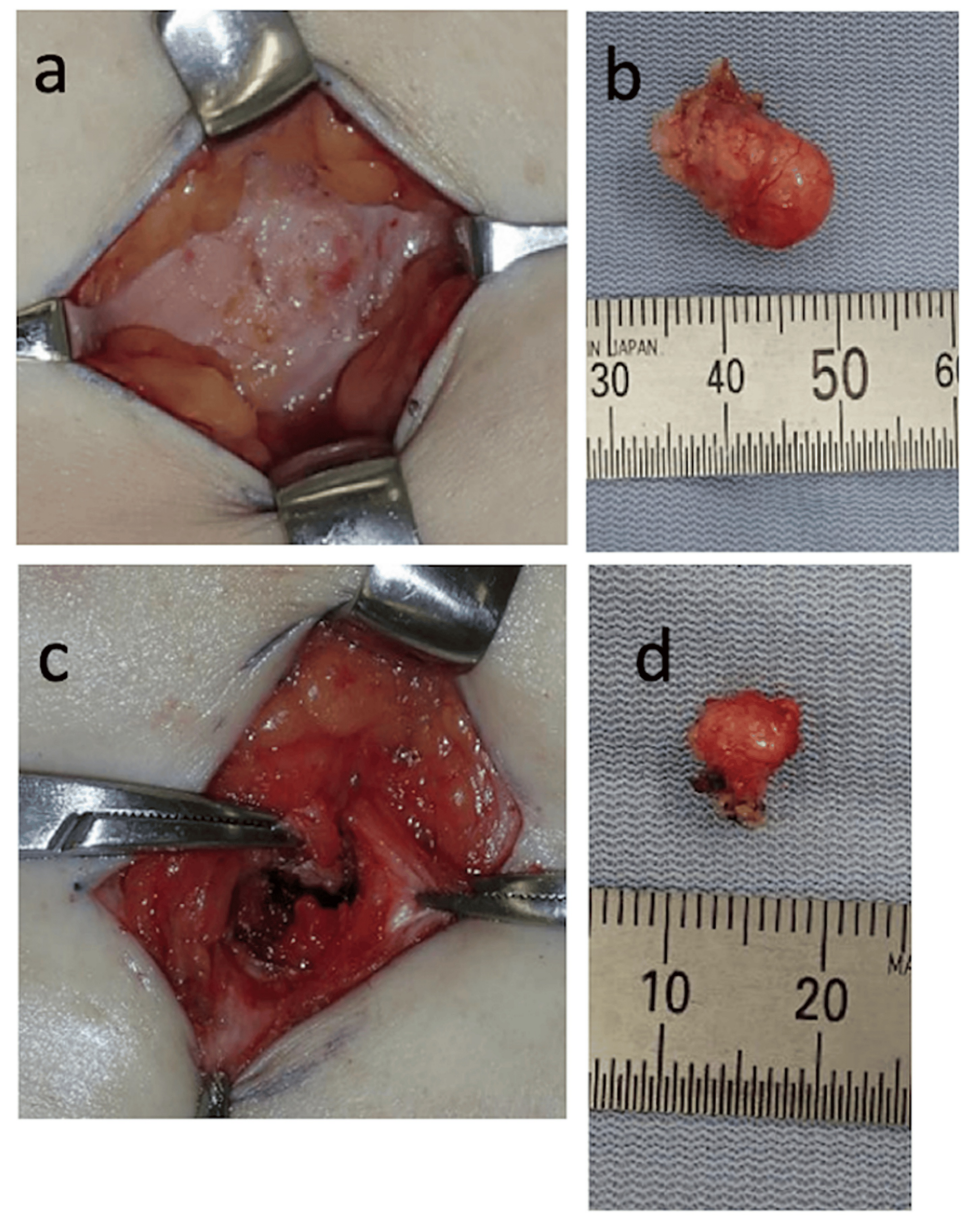
(a) The tumor located at subcutaneous. (b) Excised mass from the subcutaneous. (c) The tumor located under the rectus abdominis. (d) Excised mass removed from the subabdominal wall.

**Figure 3:**
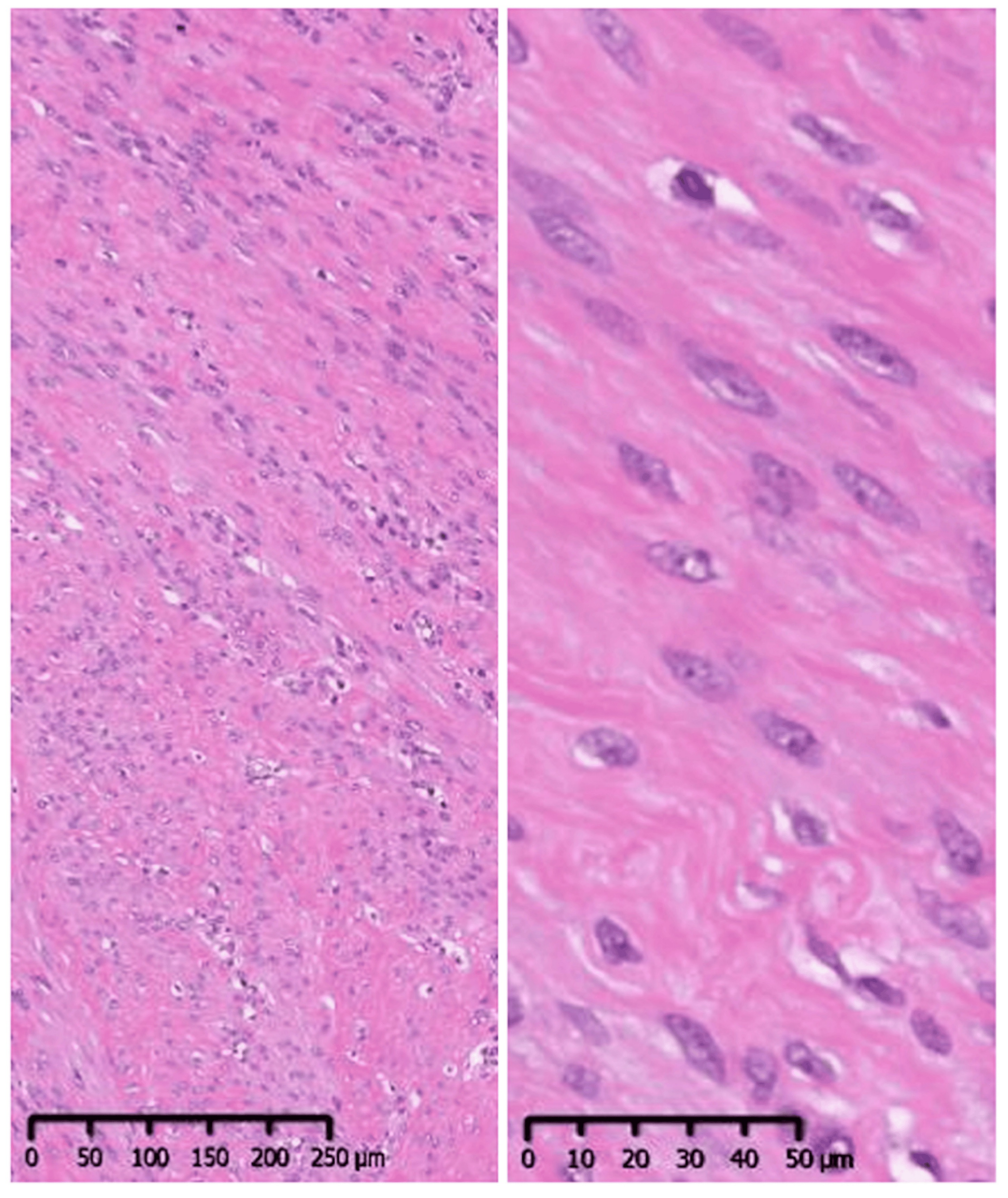
Histologic findings of the excised tumor. Hematoxylin and eosin staining is shown.

**Figure 4:**
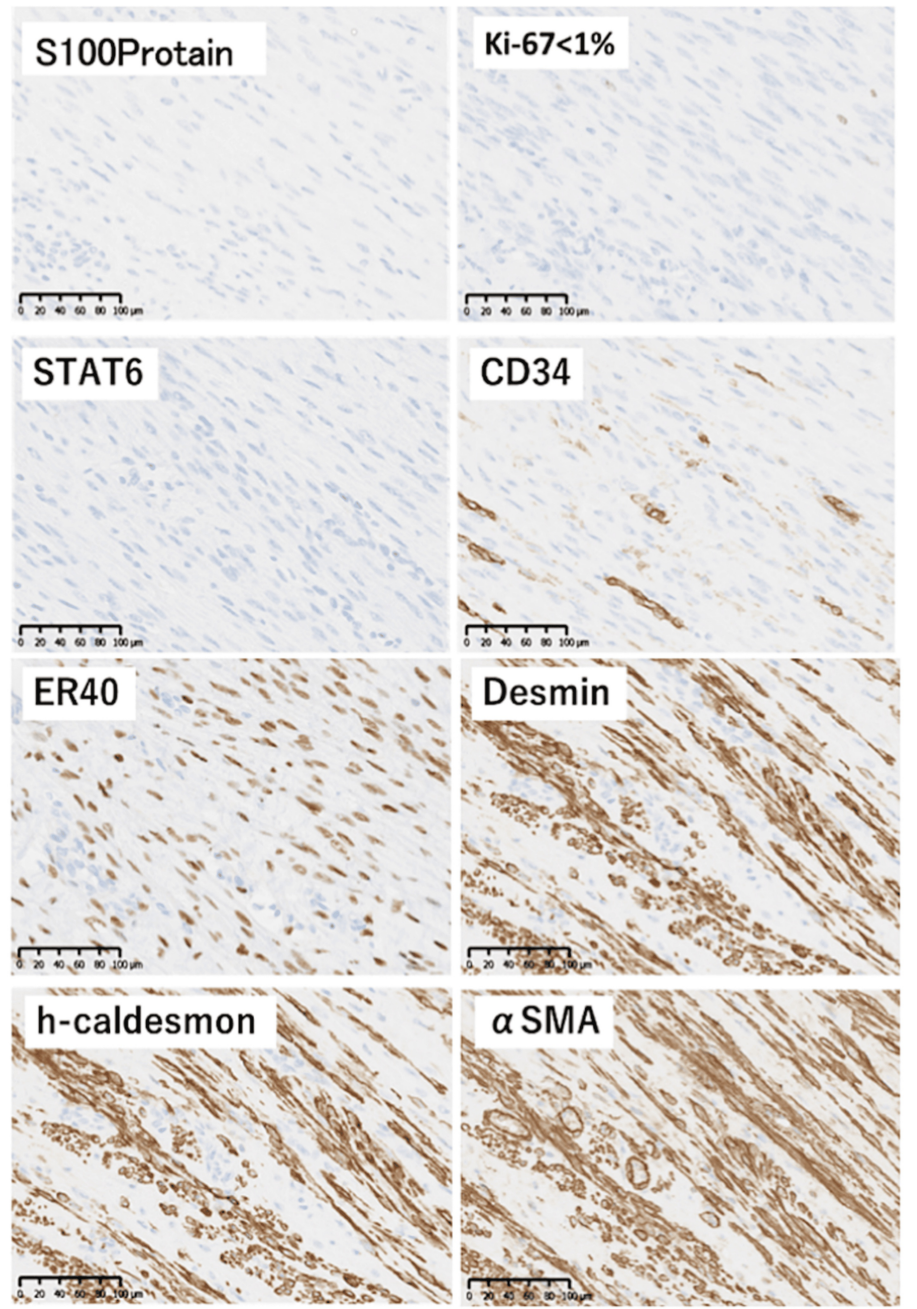
Immunostaining showing negative results (S100, Ki67, STAT6, CD34) and positive results (ER40, desmin, h-caldesmon, α-SMA).

Contrast-enhanced magnetic resonance imaging (MRI) showed low signal intensity on T1-weighted (Figure 1a) and T2-weighted (Figure 1b) images. Two tumors, measuring 10 mm in diameter in the subcutaneous tissue and 6 mm in diameter in the posterior rectus abdominis, were observed. Resection was performed under general anesthesia, and masses enveloped by scar tissue in the subcutaneous fat layer and posterior rectus abdominis were present in the right lower abdomen. The scar corresponded to the port site of the previous surgery. The excised masses measured 10 x 10 mm (Figure 2a) and 6 x 6 mm (Figure 2b), respectively. Histopathological examination with hematoxylin-eosin staining revealed a bundle-like proliferation of acidophilic spindle-shaped cells with no mitotic figures or necrosis (Figure 3). Immunostaining was negative for S100, Ki67, STAT6, and CD34 (Figure 4), confirming no malignancy, and positive for estrogen receptors (ER40), desmin, h-caldesmon, and α-smooth muscle actin (αSMA) (Figure 4), diagnosing an estrogen receptor-expressing leiomyoma of muscle origin. 

Based on the patient’s medical history, histopathological features, and positive signals of ER40, αSMA, and h-caldesmon, we diagnosed the patient with iatrogenic parasitic leiomyoma. On postoperative day two, she had a good recovery in her operated site without hematoma, infection, or inflammation and was discharged home. Two months later, she had no symptoms in the outpatient follow-up.

## Discussion

Iatrogenic parasitic leiomyoma is thought to develop when minced tissue fragments induce angiogenesis in adjacent organs, although the mechanisms involved in their localization and function are unclear [2]. Laparoscopic power morcellators were uncritically used to slice leiomyomas up until 2014 [7]. Since the present patient had a history of undergoing laparoscopic myomectomy in 2014 using a morcellator, it is strongly suggested that the remaining fragment developed into a parasitic leiomyoma. Van der Meulen et al. [3] reported that parasitic leiomyoma is estimated to occur after 0.20-1.25% of laparoscopic myomectomies, and the overall incidence of parasitic myomas after laparoscopic morcellation is 0.12-0.95%. It is unclear why parasitic leiomyomas develop in only a few patients who undergo laparoscopic myomectomy or hysterectomy. Several investigators have reported that genetic variations were associated with parasitic leiomyoma [10,11]. Furthermore, other factors that increase the risk of this complication include associated stromal cells, such as myoma-derived fibroblasts or microvascular endothelial cells, which could account for tumor formation by providing a supportive tumor stroma or a microvascular network [12]. Exposure of fragments to steroid hormones and growth factors may play a role in the development of leiomyoma [13]. Large-scale prospective research is needed to determine the incidence and risk factors for parasitic leiomyomas after laparoscopic myomectomy or hysterectomy using power morcellation.

Estrogen receptor- and progesterone receptor-positivity are specific for uterine leiomyomas and may be helpful in diagnosing parasitic leiomyoma [14]. Due to the sex hormone dependence of this benign tumor, hormonal therapy with gonadotropin-releasing hormone agonists, progesterone antagonists, selective estrogen receptor modulators, and aromatase inhibitors has been considered effective, and some studies have reported success with hormonal therapy [15]. Since the sizes of masses in this case were relatively small and immunohistochemical analysis showed high expression of estrogen receptors (ER40), hormonal therapy could be an effective treatment.

Symptoms generally include lower abdominal pain, mass sensation, and painful intercourse; excessive menstrual periods and painful urination have also been reported [16]. In some cases, iatrogenic parasitic leiomyoma is asymptomatic and found incidentally. The reported locations of iatrogenic parasitic leiomyomas include the pelvis, gastrointestinal tract, greater omentum, port entry sites, and peritoneum [16].

The present patient complained of a subcutaneous mass in the lower right abdomen without pain. The tumors were located under the skin and rectus abdominis muscle, corresponding to the scar at the port site by the morcellator in the right lower abdomen. These findings suggest that some dispersed tissue from morcellation may become implanted and persist as parasitic leiomyoma. T1- and T2-weighted axial MRI showed both tumors with low signal intensity. There was no doubt that these masses were equivalent to iatrogenic parasitic leiomyoma. There is no established treatment. However, the leiomyoma should be removed to rule out unexpected malignancy and its diagnosis confirmed by histological examination. An iatrogenic parasitic leiomyoma is a benign tumor and has a good prognosis with resection.

According to FDA warnings, methods for reducing the risk of iatrogenic parasitic leiomyoma after morcellation have been introduced; these include thoroughly flushing the abdominal cavity with a saline solution and performing in-bag morcellation [5,6]. Contained morcellation in an endoscopic bag may reduce fragment dispersion [6]. Although the number of patients with this disease is expected to decrease in the future, the efficacy of tissue contamination by in-bag morcellation is controversial regarding the non-negligible remnants of microscopic cells [17]. We cannot draw any conclusions at this point. More research on the subject needs to be undertaken. When a physician diagnoses a female patient with an abdominal mass, who has previously undergone laparoscopic myomectomy, iatrogenic parasitic leiomyoma should be considered as a differential diagnosis. Particularly, a careful search for the formation of parasitic leiomyoma in the intraperitoneal region is required, occasionally in cooperation with obstetricians and gynecologists.

## Conclusions

Iatrogenic parasitic leiomyoma likely results from the post-morcellation growth of missed uterine tissue fragments. Clinicians often encounter difficulty in diagnosing tumors preoperatively due to their rarity, aberrant locations, and inexperienced presentations. Our plastic surgeons have rare experience with iatrogenic parasitic leiomyoma after laparoscopic myomectomy using the power morcellator. The present report confirms the correlation between laparoscopic myomectomy of uterine leiomyoma and the onset of some complications such as parasitic leiomyomas in the subcutaneous tissue of the lower abdomen and posterior rectus abdominis eight years later. The excised parasitic leiomyoma revealed characteristics of port-site implantation. Physicians must ensure thorough inspection and washing of the abdominal cavity during uterine fibroid surgery using morcellation to mitigate the risk of iatrogenic parasitic leiomyoma.
